# The Synergistic and Opposing Roles of ω-Fatty Acid Hydroxylase (*CYP4A11*) and ω-1 Fatty Acid Hydroxylase (*CYP2E1*) in Chronic Liver Disease

**DOI:** 10.17352/gbmg.000003

**Published:** 2024-10-11

**Authors:** James P Hardwick, Victor Garcia

**Affiliations:** 1 Department of Integrative Medical Sciences Liver focus group, Northeast Ohio Medical University, 4209 State Route 44, Rootstown, Ohio 44272, USA; 2 Department of Pharmacology, New York Medical College, 15 Dana Road, Basic Science Building, Rm. 530, Valhalla, NY 10595, USA

## Abstract

Cytochrome P450 fatty acid hydroxylase consists of members of the CYP4 family that ω-hydroxylate fatty acids and the *CYP2E1* that ω-1 hydroxylates fatty acids. Although ω and ω-1 hydroxylation of fatty acids have been thought to play a minor role in fatty acid metabolism (less than 20%), it plays a vital role in excess liver fatty acids overload seen in fasting, diabetes, metabolic disorder, and over-consumption of alcohol and high-fat diet. This pathway provides anabolic metabolites for gluconeogenesis, succinate, and acetate for lipogenesis. The *CYP4A* and *CYP2E1* genes are activated in fasting and several metabolic disorders, suggesting a synergistic role in preventing fatty acid-induced lipotoxicity with the consequence of increased liver cholesterol and lipogenesis leading to increased Lipid Droplet (LD) deposition. During the progression of Metabolic Dysfunction-associated Steatotic Liver Disease (MASLD), activation of Phospholipase A2 (PLA_2_) releases arachidonic acid that *CYP4A11* and *CYP2E1* P450s metabolize to produce 20-hydroxyeicosatetraenoic acid (20-HETE) and 19-HETE, respectively. These metabolites have opposing roles in the progression of MASLD and chronic liver disease (CLD). This report discusses the synergistic role of the CYP4A and *CYP2E1* P450s in the metabolism of saturated and unsaturated fatty acids and their opposite physiological role in the metabolism of Arachidonic Acid (AA). We finally discuss the role of ethanol in disrupting the synergistic and opposing roles of the *CYP4A* and *CYP2E1* genes in MASLD and CLD.

## Introduction

The Human cytochrome P450 family consists of 57 individual P450s that metabolize drugs and environmental toxins. However, these cytochrome P450s also have a pivotal role in metabolizing endogenous molecules, including vitamins, cholesterol, bile acids, steroids, fatty acids, and bioactive eicosanoids [1]. The eicosanoid arachidonic acid is converted to pro-inflammatory leukotrienes that Human CYP4F family members inactivate, while pro-inflammatory 20-HETE is synthesized from arachidonic acid by *CYP4A11*. The 20-HETE eicosanoid is a high-affinity ligand for the GPR75 receptor that activates the NFκβ pathway, leading to inflammation [2]. GPR75 activation by 20-HETE causes vasoconstriction by activating Angiotensin II [3], Furthermore, 20-HETE has been implicated in activating liver stellate cells and fibrosis [4]. A recent report from a large-scale human exome study revealed that GPR75 loss-of-function variants are associated with lower body fat and BMI [5]. In mice, inactivation of GPR74 prevents dietary-induced obesity, and loss of function protects against fatty liver disease [6].

*CYP4A11* is a highly polymorphic gene with overexpression in humans that increases 20-HETE serum levels associated with hypertension and plaque in patients with ischemic stroke [7]. The expression of *CYP4A11* and 20-HETE levels has not been analyzed in patients with Metabolic Dysfunction-associated Steatosis Liver Disease (MASLD). Unfortunately, there have been no studies on the 20-HETE-producing *CYP4a12* in mice. *CYP4a14* ^−/−^ mice resist hypertension and high fat diet-induced fatty liver disease even though *CYP4a14* P450 does not metabolize arachidonic acid to 20-HETE [8]. It is suggested that stressors such as fasting or a high-fat diet activation of *CYP4a14* increase the level of *CYP4a12* and 20-HETE, leading to increased hypertension and diet-induced fatty liver disease [9].

In contrast to the ω-hydroxylation of arachidonic acid to 20-HETE, the ethanol-inducible *CYP2E1* metabolizes arachidonic acid to ω-1 arachidonic metabolite 19-HETE [10]. *CYP2E1* P450 has been implicated in alcoholic-associated liver disease through its generation of Reactive Oxygen Species (ROS), leading to lipid peroxidation. However, the role of 19-HETE produced by *CYP2E1* P450 has not been explored in association with alcoholic-associated or metabolic-associated fatty liver disease. 19-HETE is a potent prostacyclin receptor agonist that inhibits platelet aggregation, angiotensin II-induced hypertension, and fibrosis when activated while inducing vasodilation [11,12]. In this review, we explore the opposite and contrasting roles of *CYP4A11* ω-hydroxylated 20-HETE, and the *CYP2E1* ω-1 hydroxylated 19-HETE in the context of chronic liver disease.

Although *CYP4A11* 20-HETE and *CYP2E1* 19-HETE have opposing physiological roles, both cytochrome P450s have a synergistic and similar role in the metabolism of fatty acids. Hydroxylated fatty acids are sequentially metabolized by alcohol and aldehyde dehydrogenases to Dicarboxylic Acid (DCA), transported into the peroxisome, and chain-shortened by β-oxidation. Successive rounds of peroxisome β-oxidation produce shorter chain DCA, which is either transported to mitochondria for complete β-oxidation or functions as anaplerotic metabolites for gluconeogenesis or lipogenesis, resulting in Lipid Droplet (LD) formation and hepatic steatosis.

In this review, we discuss the opposing and contrasting roles of *CYP4A11* and *CYP2E1* in the metabolism of arachidonic to 20-HETE and 19-HETE, which have opposing physiological roles through activation of GPR75 and prostacyclin (IP) receptors, respectively. We also explore the similar and synergistic role of the *CYP4A11* and *CYP2E1* P450s in the metabolism by analyzing the catalytic activity of these P450s in the metabolism of arachidonic and saturated fatty acids. We identify the possible role of CYP P450 arachidonic acid and fatty acid metabolites in diabetic hyperglycemia, LD formation in hepatic steatosis, and their functional role in hypertension through their effect on hepatic hepatocytes, stellate, and endothelial cells. Finally, we discuss the function of *CYP4A11* and *CYP2E1* saturated fatty acid metabolites serving as anaplertoic metabolites for fatty acid and cholesterol synthesis leading to increased lipid droplet formation in hepatic steatosis. We also detail the role of the *CYP4A11* and *CYP2E1* arachidonic metabolites and their possible functional role in portal hypertension in hepatic cirrhosis and suggest alteration in these pathways may be therapeutic targets in the treatment of chronic liver disease.

## Background

### Omega and Omega-1 cytochrome P450 hydroxylase in fatty acid metabolism

**Figure 1:**
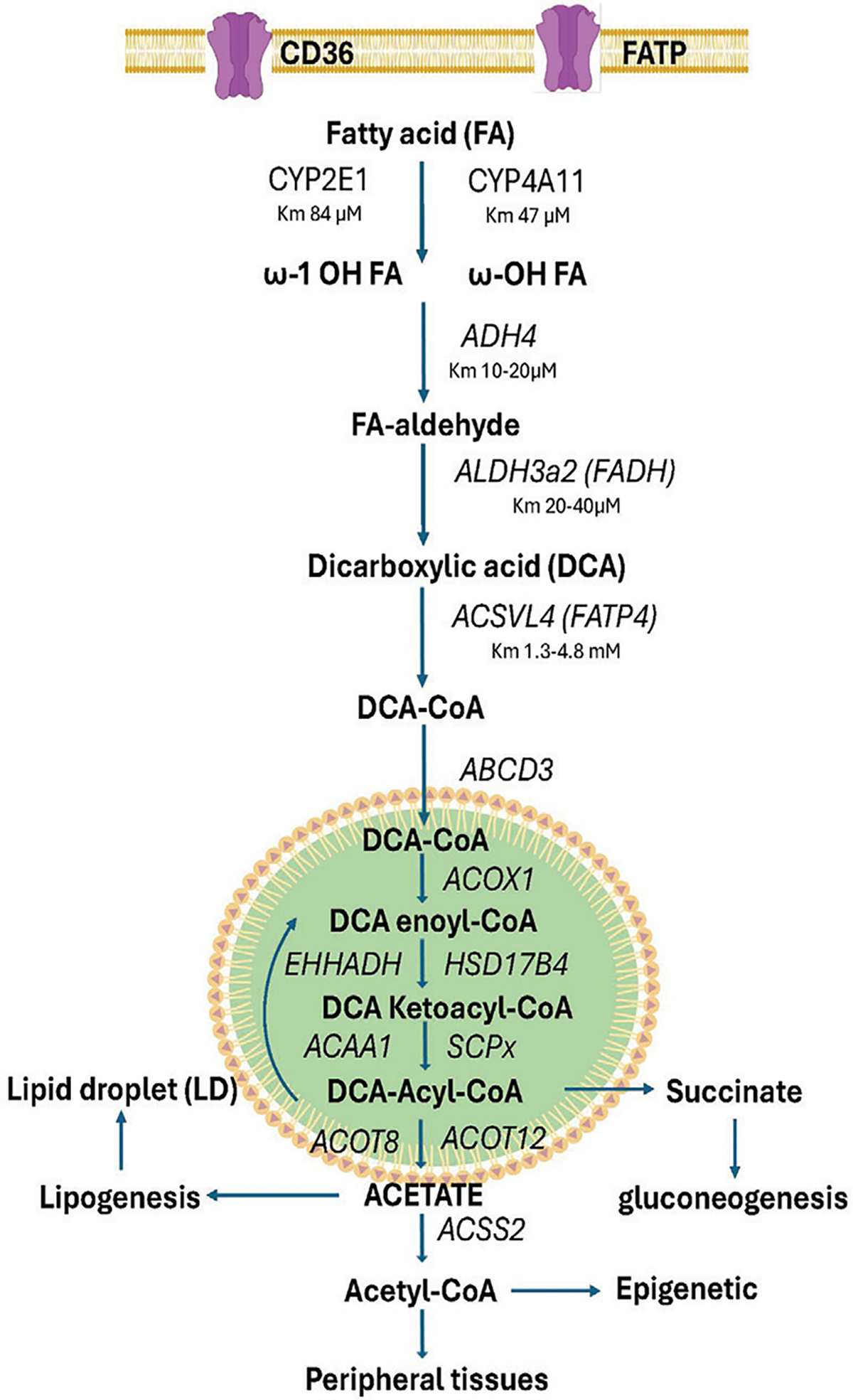
The synergistic role of Omega P4504A11 and Omega-1 P4502E1 hydroxylation of saturated fatty acids and further metabolism by peroxisome β-oxidation. CYP4A11 P450 has a lower Km for saturated fatty acids than CYP2E1 P450. Microsomal alcohol dehydrogenase (ADH4) and Aldehyde Dehydrogenase (ALDH3a2) have similar Km values for hydroxylated fatty acids. Also, the oxidation rates of their enzymatic activities are linked to the metabolism of hydroxylated fatty acids. Dicarboxylic Acid (DCA) accumulates in the cytosol because Acyl-CoA synthetase (ACSVL4) has a high Km. ABCD3 transports DCA into mitochondria to be metabolized by Acyl-CoA oxidase 1 (ACOX1) and the peroxisomal bifunctional hydratase/dehydrogenase (EHHAH-HSD17B4). Acetyl-CoA is released from Acyl-CoA by acetyl-CoA acyltransferase (ACCA1) or SCPx thiolase. Acetate is formed from acetyl-CoA by acyl-CoA thioesterase 12 (ACOT12) or acyl-CoA thioesterase 8 (ACOT8). The acyl-CoA, succinate is released from succinyl-CoA by ACOT4. Acetate can be used as an energy source by peripheral tissues or in hepatocytes as a substrate for fatty acid or cholesterol synthesis, resulting in Lipid Droplet (LD) formation. Mitochondria can use succinate in fasting for gluconeogenesis. Both succinyl-CoA and acetyl-CoA are substrates that may epigenetically modify chromatin structure through epigenetic compartmentalization of epigenetic metabolites.

Fatty acid omega (ω) and omega-1 (ω-1) cytochrome P450 hydroxylases are considered to have a minor role in the metabolism of fatty acids; however, their importance increases in fasting and several disease states, including diabetes [13–16], contributing up to 20% of fatty acid oxidation (Figure 1). *CYP4A11* P450 binds saturated lauric acid (C12:0) with a Km of 4.7 μM, and Kcat of 7 min^−1^, while *CYP2E1* binds with a Km 84μM and Kcat of 3.8 min^−1^ [10,17], far below the plasma levels of palmitic and stearic acid (0.03–4 mM) observed in human plasma. Alcohol dehydrogenase ADH4 binds fatty acids with a Km of 10–20 μM and cytosolic aldehyde dehydrogenase ALDH3A2, also known as Fatty Aldehyde Dehydrogenase (FALDH) binds fatty acids with Km of 20–40 μM [18]. It is of interest that, unlike other aldehyde dehydrogenases, ALDH3A2 and 3B2 use NADP^+^ as a cofactor and, therefore, can regenerate NADPH for cytochrome P450 metabolism of the fatty acid substrates. In addition, the tight association of ADH and ALDH3A2 in microsomes makes for an efficient system to eliminate reactive aldehydes. The microsomal long-chain Fatty Acid Transport Protein 4 (FATP4, ACSVL4) converts the Dicarboxylic Acids (DCAs) to their respective CoAs and binds C16:0 to C24:0 with Km of 13 to 4.8 mM [19].

The DCA-CoA is transported into the peroxisome by the 70-KDa peroxisomal membrane protein (PMP70, ABCD3) [20,21]. Acyl-CoA oxidase (ACOX1) catalyzes the first step in peroxisome β-oxidation while the L- and D-bifunctional protein enoyl-CoA hydratase (EEHADH) and 3-hydroxyacyl CoA dehydrogenase 17B4 (HAD17B4), respectively, catalyze the hydratase/dehydrogenase steps in the β-oxidation of fatty acids with EEHADH having a 3-fold lower affinity for DCA-CoA [22]. The thiolase, sterol carrier protein x (SCPx), and acetyl-CoA acyltransferase 1a (ACAA1a), with ACAA1b functions as the major 3-keto-CoA thiolase B for the hydrolysis of DCA-CoA. Additionally, the rate of β-oxidation of DCAs decreases with short chain-length DCAs, suggesting a significant reason for the appearance of adipic (C6), sebacic (C10), and suberic (C8) DCAs in the urine [23–25]. Shorter chain DCAs are produced and transported into the cytosol, functioning as anaplerotic intermediates in metabolism [26]. One of the shorter-chain DCs that contribute to anaplerotic reactions is succinyl-CoA, which is converted by ACOT4 thiolase to succinate [27,28] that functions to replenish the TCA cycle and as a gluconeogenic metabolite [29]. Also, acetyl-CoA is converted by ACOT12 and ACOT8 thiolase to produce acetate [30,31] which can be used as an energy source in peripheral tissues or an anaplerotic metabolite for lipogenesis and cholesterol synthesis [1,32,33], resulting in increased Lipid Droplet (LD) formation in the liver [33,34], It will be interesting to determine whether fatty acid or DCA is the preferred source of acetate used in fatty acid synthesis and LD formation.

The ω and ω-1 oxidation of medium and long-chain fatty acids has received much interest since it has long been thought that activating this pathway in stressful conditions of fasting, diabetes, and high-fat diets prevents fatty acid toxicity. DCAs are potent uncouplers of mitochondrial respiration [35,36]. DCA increases in many mitochondrial FAO disorders where ω-oxidation and peroxisome β-oxidation increase DCA excretion [29]. This is a compensatory mechanism in which DCA metabolism by peroxisome β-oxidation produces anaplerotic metabolites, succinate, and acetyl-CoA, that increase the mitochondrial TCA cycle. Elevated cytosolic acetate from the metabolism of DCA increases fatty acid and cholesterol synthesis, leading to enhanced hepatic triglyceride synthesis and lipid droplet formation. Determining if saturated fatty acid or DCA-derived succinate and acetate induce changes in the epigenetic metabolic program will be important [37].

Both urinary and plasma levels of DCA increase in several disease states that include diabetes [38], MASLD [39], inborn error Fatty Acid Oxidation (FAO) defect [40,41], celiac disease [42], dicarboxylic aciduria [25], and Reye’s syndrome [36]. In each of these conditions decreased mitochondrial oxidation is most likely compensated by the ω-hydroxylation and peroxisome β-oxidation of FAs. DCAs have been proposed as an alternative energy source in several disease states by providing anaplerotic intermediates of the TCA cycle. Oral administration of sebacic acid to Type II Diabetics (T2DM) improved glycemic control, improved insulin sensitivity, and reduced hepatic gluconeogenesis and glucose output. [43] In addition, dodecanedioic acid (C12-DCA) reduced muscle fatigue in T2DM patients. Recently, the hydroxylated C13-DCA, bempedoic acid, has been approved to lower LDL by inhibiting ATP-citrate lyase, reducing cholesterol and fatty acid synthesis (FAS), and activating AMP-protein Kinase (AMPK) to suppress gluconeogenesis and lipogenesis [44]. Administration of dodecanedioic acid DCA12 prevented acute kidney injury. Dietary 12-DCA reduced body fat, and fatty liver, and improved glucose tolerance in mice [45]. Increased levels of DCA are excreted in human urine under medium-chain triglyceride (MCT) feeding [25], which is used in the treatment of Alcohol-associated Liver Disease (ALD) [46] and as a nutritional supplement. Furthermore, in mice with hepatoblastoma, a deficiency of DCA catabolism with the administration of Dodecanedioic Acid (DDDA) led to hepatoblastoma necrosis and significantly longer survival than mice on standard diets [47].

### Functional role of human CYP4 ω-hydroxylase in MASLD

A high-fat diet induces the ω-oxidation of fatty acids and increases the expression of *CYP4A11* in HepG2 human hepatoma cells [1,48]. RNA seq database analysis of patients with MASLD revealed increased *CYP4A11* and *CYP4A22* mRNA in steatosis and MASH [32]. In addition, the *CYP4F2* and *CYP4F3a* genes were increased in patients with steatosis, MASH, and cirrhosis. The CYP4F8 prostaglandin hydroxylase and *CYP4F11* mRNA levels increase in steatosis, MASH, and advanced hepatocellular carcinoma. In human patients, the *CYP4F12, CYP4F22*, and *CYP4V2* mRNA levels increase in steatosis and MASH, while the levels of *CYP4B1*, *CYP4X1*, and *CYPZ1* mRNAs decrease in steatosis and MASH [32] while the mRNA for these genes increases in HCC. In these RNA seq datasets, GSE13251 and GSE114564, GSE114564 and GSE 113564, PNPLA3 and MBOAT7 mRNA levels increase in steatosis, MASH, and advanced HCC. In contrast, mRNA levels of *CYP2E1* and *CYP3A4*, major drug metabolizing genes, decrease in steatosis, MASH, and HCC. These data indicate that members of the CYP4 gene family members contribute to the progression of MASLD through increased ω-hydroxylation of different chain-length FAs and bioactive eicosanoids. Fasting induces hepatic lipid accumulation through peroxisome β-oxidation of various DCAs [33], suggesting that increased lipolysis of these FAs elevates acetate levels, hepatic lipogenesis, and Lipid Droplet (LD) formation [1], leading to inflammatory MASH [49]. In human HepG2 cells, incubation with 1 mM FFA increased the expression of *CYP4A11* 3-fold and the levels of triglycerides 10-fold, leading to an elevation of 2.5-fold in Reactive Oxygen Species (ROS) and lipid peroxidation 3-fold [48]. Over-expression of *CYP4A11* increases expression of Tumor Necrosis Factor (TNF), Interleukin-1β (IL-1β), and interleukin 6 (IL-6) through activation of the NF-κβ signaling pathway. It will be essential to determine if the DCA levels increase and whether acetyl-CoA or acetate levels elevate since these latter molecules exhibit both anaplerotic and epigenetic effects on the progression of MASLD. It will be essential to determine if peroxisome compartmentalized acyl-CoA metabolism has a role in chromatin regulation [50] since it has recently been shown that ALDH1A3-acetaldehyde metabolism potentiates melanoma transcription heterogeneity [51,52].

In *Cyp4a14*^−*/*−^ mice fed a high-fat diet, 20-HETE induces obesity and insulin resistance since the 20-HETE antagonist mediates [53], prevented hyperglycemia and hyperinsulinemia. In contrast, *Cyp4a14*^−*/*−^ mice are resistant to hepatic steatosis and fibrosis, while Ad-*Cyp4a14* mice exhibited an increase in hepatic TG and LD formation due to enhanced uptake of fatty acid by elevated expression of CD36 [8]. Angiotensin II initiates renal fibrosis and increases *cyp4A14* expression mediated by the Mitogen-activated Protein Kinase (MAPK) pathway. *Cyp4a14*^−*/*−^ mice are resistant to Ang II-induced renal fibrosis. Moreover, *Cyp4a14*^−*/*−^ mice also resist bile duct ligation-induced cholestatic liver fibrosis [54]. Several of these studies attribute insulin resistance, obesity, and fibrosis to *Cyp4a14*-mediated increased production of 20-HETE. However, *Cyp4a14* p450 does not metabolize AA to 20-HETE [55]. The mouse has four major *Cyp4a isoforms, Cyp4A10, Cyp4A14*, and *Cyp4a12a*, male-specific, and *Cyp4a12b*, female-specific with five minor forms, including *Cyp4a29*, *Cyp4a30b, Cyp4a31, and Cyp4a32* [56]. *Cyp4a14*^−*/*−^ mice exhibit increased plasma androgens. Androgen increases *Cyp4a12* expression in the kidney, increasing 20-HETE formation in kidney microsomes [9]. This compensatory increase in *Cyp4a12* P450 but not 4a10 P450 in *Cyp4a14*^−*/*−^ mice further points to *Cyp4a12* P450’s role in AA metabolism to 20-HETE. It will be important to determine if hepatic levels of 20-HETE increase in the *CYP4a14*^−*/*−^ mice since *Cyp4a14*^−/−^ female mice do not show an increase in *Cyp4A12* in kidney microsomes.

### Functional role of *CYP2E1* in MASLD and alcohol-associated liver disease

Alcohol-induced MASLD progression is related to the amount and duration of alcohol consumption [57,58], with excessive alcohol consumption leading to an increase in *CYP2E1* activity, producing ROS causes Endoplasmic Reticulum (ER) stress and mitochondrial dysfunction that negatively inhibit FA oxidation [59]. *CYP2E1* contributes to oxidative stress and steatosis in chronic alcohol-exposure models and MASLD [40] by increasing lipid peroxidation and decreasing levels of antioxidants, superoxide dismutase, glutathione peroxidase, and aldehyde dehydrogenase, leading to elevated levels of protein carbonylation, nitration, phosphorylation and glycation that contribute to increased insulin resistance and impaired glucose tolerance [60]. *CYP2E1* P450 is a high-spin cytochrome that raises the redox potential and facilitates the generation of ROS [61,62] Despite using the P450 2E1 (*CYP2E1*) inducer isoniazid, there was no increase in F2-isoprostane production, the gold standard for ROS determination [63]. Also, the *Cyp2e1*^−*/*−^ mice had similar levels of urinary isoprostanes as the wild-type animals, indicating that microsome *CYP2E1* induction by ethanol or isoniazid does not increase ROS [63]. However, mitochondrial *CYP2E1* did show enhanced isoprostane levels after ethanol treatment [64]. *CYP2E1* protein is found in both the endoplasmic reticulum and mitochondria. However, the role of the mitochondria in alcohol-associated or non-alcoholic MASLD is uncertain [64,65]. Similar to the ER-*CYP2E1,* the mt*CYP2E1* increases levels of ROS and mitochondrial 3-nitrotyrosine and 4-hydroxynonenal protein adducts and decreased mitochondrial aconitase activity and mitochondrial membrane potential [66]. The distribution of the *CYP2E1* in microsomes and mitochondria in the human liver may account for individual differences in ethanol toxicity and the initiation of alcohol-Associated Liver Disease (ALD) [67].

*CYP2E1* P450 can ω-1 hydroxylate saturated and unsaturated fatty acids [10,68,69]. *CYP2E1* P450 metabolizes lauric and myristic acids to their respective ω-metabolites with turnover numbers of 3.8 and 2.4 min^−1^, respectively, while *CYP4A11* P450 metabolizes lauric and myristic acid with turnover numbers of 7.3 and 2.1 min^−1^, respectively [17]. *CYP2E1* and *CYP4A11* P450 bind lauric acid with similar Km values (5.8 and 4.7 μM, respectively), indicating competition in the metabolism of saturated fatty acid substrates. Both alcohol dehydrogenase ADH4 has a similar affinity for both ω and ω-1 hydroxylated fatty acids (ADH, Km 10–20 μM) in comparison to the oxidation of ethanol (2–40 mM), indicating that ethanol is not the physiological substrate for *CYP2E1* P450 under normal conditions. [70] In addition, aldehyde dehydrogenase (ALDH3A2, Km 4–19 μM) [71] confirms the importance of the ω and ω-1 pathways in the metabolism of fatty acids. RNA-seq database analysis revealed that *CYP2E1* expression is decreased during the progression of MASLD [32], suggesting that the level of 19-HETE decreases and 20-HETE increases. This indicates that the ω-hydroxylation pathway has a significant role in the progression of MASLD.

Liver Sinusoidal Endothelial Cells (LSECs) can metabolize ethanol, and ethanol can increase *CYP2E1* levels that produce acetaldehyde and acetyl-CoA, a substrate for protein acetylation and lipid synthesis. However, the *CYP2E1* ω-1 hydroxylation of fatty acid and peroxisomal β-oxidation would contribute to the acetyl-CoA pool. Ethanol increases heat shock protein 90 (Hsp90) acetylation and decreases its interaction with endothelial Nitric Oxide Synthetase (eNOS), resulting in decreased production of eNOS-derived Nitric Oxide (NO) [72]. The eNOS-derived NO-signaling [73] is a critical player in LSEC function through the activation of Vascular Endothelial Growth Factor (VEGF) that maintains LSEC fenestrae and prevents capillarization [74,75] The activity of eNOS is regulated by its interaction with several proteins, including the G-Coupled Protein Receptor (GCPR) β-Arrestin 2 and Hsp90. An increase in the acetyl-CoA pool results in Hsp90 acetylation, decreasing its interaction with eNOS and producing eNOS-derived NO [72]. Overexpression of the histone deacetylase 6 (HDAC6), which deacetylates Hsp90, increases the association of Hsp90 with eNOS, leading to elevated NO production [76]. Also, eNOS activity and NO production are enhanced by its interaction with β-arrestin-2 [77]. It is currently unknown to what extent the hepatocyte, LSEC microsome, mitochondrial *CYP2E1*, or its metabolites contribute to endothelial dysfunction in ALD and MASLD. Moreover, it will also be important to determine whether *CYP4* P450s are expressed in LSEC.

### Role of *CYP4A11*- and *CYP2E1*-mediated fatty acid metabolism in MASLD

The synergistic role of *CYP2E1* and *CYP4A11* in the saturated and unsaturated fatty acids metabolism indicates their vital role in fasting, diabetes, and the progression of MASLD. Numerous studies have shown that *Cyp2e1*^−*/*−^ knock-out mice are resistant to hepatic steatosis, steatohepatitis, and liver fibrosis by reducing fatty acid synthesis [78], decreasing ROS, and free radical production, hindering lipid peroxidation while increasing the expression of antioxidant genes and hepatic levels of glutathione [79,80] Both insulin resistance and hyperinsulinemia have a crucial role in hepatic fat accumulation and reflect common conditions of ALD and MASLD. *Cyp2e1*^−*/*−^ mice are protected from high-fat-induced insulin resistance [60,81] with insulin, which is known to decrease *Cyp2E1* expression [82]. *Cyp2e1*^−*/*−^ mice expressing the human *CYP2E1* transgene show increased hepatic steatosis, oxidative stress, insulin resistance, and liver injury [83]. It is unknown whether insulin resistance in MASLD increases *CYP2E1* by ketone bodies that stabilize *CYP2E1* P450 and prevent its degradation by the ubiquitin-dependent proteasome system.

The interplay of the *CYP2E1* and *CYP4* genes in steatohepatitis is evident in *Cyp2e1*^−*/*−^ mice administered a methionine choline-deficient (MCD) diet as a model of MASLD. The *Cyp2e1*^−*/*−^ mice neither prevented nor inhibited lipid peroxidation but induced an upregulation of the *Cyp4A10* and *Cyp4A14* genes [84], suggesting a synergistic relation between the *CYP2E1* and *CYP4A* genes in the progression of MASLD. In the obese db/db mice, inhibiting the *Cyp4A14* gene reduces ER stress, apoptosis, insulin resistance, and steatosis [85]. The *CYP4a14*^−*/*−^ mice show attenuated hepatic steatosis and fibrosis [40.54]. Again, this reveals a close relationship between the *CYP2E1* and *CYP4A* genes in the progression of MASLD. It will be important to determine if hyperinsulinemia inhibits the expression of the *CYP2E1* gene and increases the expression of *CYP4A* genes in MASLD [86]. We analyzed the expression of the *CYP4A11*, *CYP4F2* and *CYP2E1* in patients with steatosis, steatohepatitis, cirrhosis, and HCC and found that both *CYP4A11* mRNA and protein increase in the progression of MASLD [52], while *CYP4F2*, the primary P450 in the metabolism of AA to produce 20-HETE, decreases in MASLD progression.

### An opposing role of *CYP4A11* and *CYP2E1* in the metabolism of arachidonic acid

Both the *CYP2E1* and *Cyp4A14* genes are synergistically regulated by the nuclear transcription factor erythroid factor 2 (NRF2) during fasting, diabetes, high-fat diet, and ethanol intoxication [87,88]. NRF2 activates several antioxidant genes [89] and Nrf2^−/−^ mice display increased high-fat-induced steatosis and steatohepatitis [90]. *Cyp2e1*^−*/*−^ and *Cyp4a14*^−*/*−^ mice resist high-fat diet-induced liver damage [8]. The observation that *Cyp2e1*^−*/*−^ mice fed an MCD diet have severe steatohepatitis with increased *Cyp4a10* and *Cyp4a14* suggests a relationship between these genes in MASLD progression. High-fat diet-induced obesity and insulin resistance in *CYP4a14*^−*/*−^ mice are believed to be due to increased expression of the *Cyp4a12a* gene that produces the vasoconstrictive 20-HETE. In the *Cyp2e1*^−/−^ mice fed the MCD diet, there was no increase in *Cyp4a12a* mRNA [84], indicating a complex synergistic or opposing role of the *CYP4A* and *CYP2E1* genes at different stages of MASLD.

The increased expression of the *CYP2E1* and *CYP4A11* genes in steatosis, with a down-regulation of the *CYP2E1* P450 and elevated *CYP4A11* in the progression of MASLD indicate the differential role of these genes. Both *CYP2E1* and *CYP4A11* proteins metabolize AA to 19-Hydroxyeicosatetraenoic Acid (19-HETE) and 20-hydroxyeicosatetraenoic acid (20-HETE), respectively, with 19-HETE having a vasodilatory effect and 20-HETE a vasoconstrictive eicosanoid [10,91,92] The 20-HETE eicosanoid is a critical player in influencing the sensitivity of the vasculature to constrictor stimuli, regulating endothelial function, influencing the renin-angiotensin system (RAS), a driver of vascular remodeling independent of blood pressure elevations, and the migration and proliferation of certain liver cells as well as metabolic syndrome and liver fibrosis [93,94,95] Mice fed a high-fat diet showed significantly higher 20-HETE/EET+DHET formation in the liver, indicating a role in the progression of metabolic syndrome [96]. In contrast, the role of *CYP2E1* in producing19-HETE in MASLD is unknown although 19-HETE protects cardiomyocytes from hypertrophy, promotes smooth muscle relaxation, and is believed to antagonize the effects of 20-HETE [97,98]. *CYP4a11* P450 has a Km of 228 μM for AA, a turnover number of 49.8 min^−1^, and a catalytic efficiency of 0.21 M^−1^ sec^−1^ [99]. In contrast, *CYP2E1* P450 has a Km of 62 μM for AA with a turnover number of 0.08 min^−1^ with a catalytic efficiency of 0.0013 M^−1^ sec^−1^, indicating the AA is metabolized to 20-HETE over *CYP2E1* synthesis of 19-HETE [10,100].

**Figure 2:**
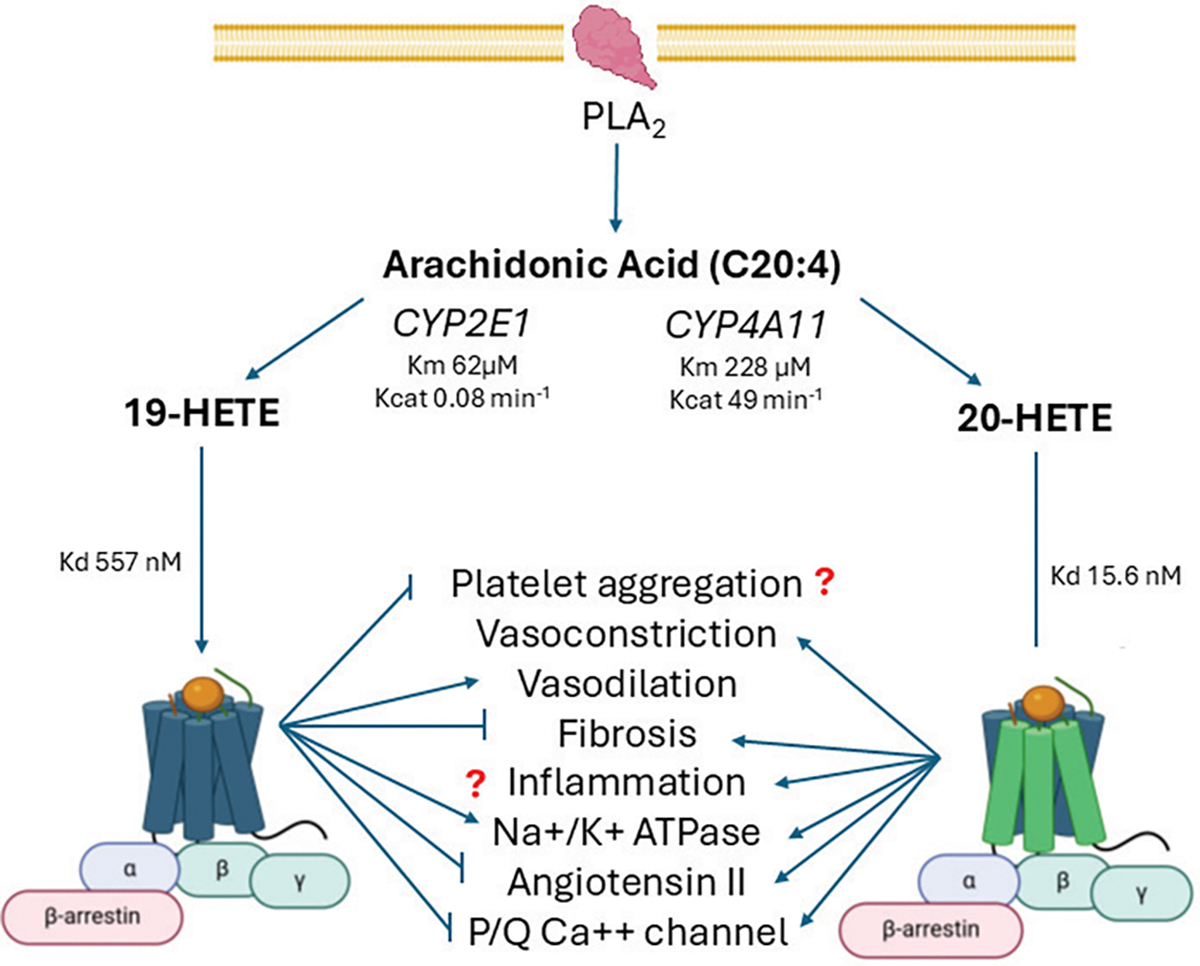
The opposing role of P4502E1 19-HETE and P4504A11 20-HETE in regulating physiological processes. During stress or inflammation, Phospholipase A2 (PLA2) releases Arachidonic Acid (AA) from membrane phospholipids. Arachidonic acid is metabolized by P4504A11 to 20-HETE or 19-HETE by P4502E1. P4502E1 has a low Km for AA (62 μM) than P4504A11 (228 μM), but P4504A11 has a high Kcat 49 min-1 than P4502E1 kcat 0.08 min-1. However, P4502E1 makes up 6% of human microsome P450 while P4504A11 represents only 1%. The prostacyclin (IP) receptor has a lower affinity for 19-HETE (Kd 567nM) than GPR75 affinity for 20-HETE (15.5 nM). The CYP2E1 or CYP4A11 P450 level and their induction by ethanol or fatty acids determine which pathway becomes more active in Chronic Liver Disease (CLD).

Both 19-HETE and 20-HETE bind specific receptors to initiate their biological effects. The 19-HETE induces vasorelaxation and inhibition of platelet activation by activating the prostacyclin (IP) receptor [91]. In contrast, 20-HETE initiates its biological effects by activating the GPR75 receptor [2,3,101]. (Figure 2). The opposing roles of *CYP2E1* 19-HETE and *CYP4A11* 20-HETE are apparent from 19-HETE’s antagonism of 20-HETE-induced vascular sensitization and hypertension [97], inhibition of the Na^+^/K^+^-ATPase [102,103] and functions to increase volume absorption in renal proximal tubules [102]. These effects are modulated by the inositol phosphate (IP) receptor coupled Gαs-mediated protein kinase A (PKA) activation that increases cAMP levels [11]. In contrast, the 20-HETE activation of the GPR75 receptor causes opposing biological effects compared to the 19-HETE-IP receptor. Activation of the GPR75 receptor by 20-HETE stimulates Gαq/11 dissociation, accumulation of IP, and binding of GPRC-Kinase Interacting Protein-1 (GIT1) that facilitates Src transactivation of endothelial EGFR [3]. Activation of endothelial EGFR by GPR75 stimulates the MAPK pathway, resulting in NF-κβ activation [2] of Angiotensin-Converting Enzyme (ACE) and the uncoupling of eNOS.

The 20-HETE/GPR75 activation of the NF-κβ pathway and suppression of endothelial eNOS have important implications for autocrine signaling in sinusoidal homeostasis in CLD, from portal hypertension to vascular thrombosis. Suppression of eNOS leads to capillarization (development of basement membrane) of the Liver Sinusoidal Endothelial Cell (LSEC) accompanied by increased vasoconstrictors, proinflammatory, profibrotic, and prothrombic factors [104,105]. NO has an anti-fibrotic role in LSEC mediated by the VEGF pathway, which is diminished by capillarization [73]. *CYP4A11*-mediated production of 20-HETE induces hepatic fibrosis via activating the TGF-β1/Smad signaling pathway [4]. Endothelial eNOS is critical to CLD [106]. 20-HETE mediated increase in plasma level of angiotensin-II is significantly elevated in patients with liver cirrhosis and is found to be a critical factor in inducing portal hypertension [75,107]. Furthermore, angiotensin-II also induces the proliferation of hepatic stellate cells, increases the mRNA level of TGF-β1 and collagen-I, and stimulates the formation of the Extracellular Matrix (ECM) [108,109] It has been shown that patients with liver cirrhosis excrete high levels of 20-HETE [110,111] It is known that 20-HETE is a weak, Cyclooxygenase (COX)-dependent vasoconstrictor of the portal circulation, and it was supposed to be involved in the pathophysiology of portal hypertension [111,112] In addition, 20-HETE was found to be involved in abnormalities related to liver diseases, particularly cirrhosis [112]. It is unknown whether 20-HETE or COX2-mediated metabolism of 20-HETE to 20-hydroxy PGH2 mediates the effects of 20-HETE in liver cirrhosis [113]. However, combined therapy with a COX2 and 20-HETE inhibitor reduces colon tumor growth, suggesting a synergistic relationship between 20-HETE and prostanoids [114]. Interestingly, MC38 colon tumor-bearing mice treated with the COX2 inhibitor Rofecoxib had increased plasma levels of 20-HETE with no increase in CYP4a expression. Elevated 20-HETE induces phosphorylation of extracellular signal-regulated kinase (ERK) 1/2 and cyclin D-1/2, suggesting that increased 20-HETE would increase cell proliferation, as seen in MC38 colorectal cells. However, treatment with Rofecoxib and CYP4 inhibitor HET0016 dramatically decreased cell proliferation. These results suggest that 20-HETE has a pivotal role in undefined cell proliferation [115]. Over 50% - 75% of AA-produced metabolites are 19-HETE and 20-HETE [116], with 20-HETE a COX-dependent vasoconstrictor of portal circulation in portal hypertension [111,112], while 11,12-EET was a vasoconstrictor in the porto-sinusoidal circulation, but functions as a vasodilator of mesenteric arterial vessels. These results suggest that 20-HETE or 20-PGG_2_/PGH_2_ causes portal vein vasoconstriction while EET induces vasodilation of mesenteric arteries resulting in increased blood flow and portal hypertension in cirrhosis. Several studies have suggested that inhibition of COX1/2 attenuates the vasoconstrictive properties of 20-HETE [113]. It has also been shown that 20-OH-PGE_2_ enhances adipocyte hypertrophy, leading to dysfunctional adipogenesis [117]. It is unknown whether 20-HETE or the metabolism of 20-HETE by either COX1 or COX2 to 20-PGG_2_/PGH_2_ have divergent effects on portal hypertension. The 20-HETE-producing cytochrome P450s are up-regulated in human cancer [118] and induce mitogenic and angiogenic responses. Combination therapies for HCC with celecoxib and sorafenib enhanced growth inhibition [119,120], and celecoxib with a tyrosine kinase inhibitor decreased proliferation and angiogenesis in HCT colon cancer cells [121]. It will be critical to determine the functional roles of 20-HETE and 20-OH PGG_2_/PGH_2_ in portal hypertension and progression of MASLD.

### Physiological role of 20-HETE and 19-HETE in disease states

The opposing autocrine and paracrine roles of 20-HETE and 19-HETE in the regulation of metabolism are evident by high-fat diet-mediated *SREBP-1a* induction of *CYP4F2* [122] that produces 20-HETE, which increases insulin secretion [11,91], leading to suppression of the *CYP2E1* [82]. Increased 20-HETE impairs insulin signaling, and its effect requires activating its receptor GPR75. In contrast, hepatocyte-specific overexpression of *CYP2E1* increased hepatic oxidative stress in the liver, fasting insulin, and histological liver damage [83]. *CYP2E1* overexpression reduced hepatic insulin signaling, decreased glycogen storage, and increased glucose synthesis. *CYP2E1* hepatic overexpression increased oxidative stress, increased systemic insulin resistance, decreased insulin signaling in the liver, and increased hepatic fat accumulation. Elevated 20-HETE contributes to HFD-induced obesity, insulin resistance, and impaired insulin signaling [53]. In contrast, Isoniazid induction of *Cyp2E1* decreased both *Cyp4A* and 20-HETE levels with an increase in 19-HETE [123]. Triple siRNA lipid nanoparticles (LNPs) targeting *Cyp2e1*, *Cyp4a10*, and *Cyp4a14* significantly ameliorated chronic alcohol-associated liver injury [124].

### Differential ethanol metabolism by fatty acid hydroxylase metabolites

Ethanol impacts mitochondrial and peroxisomal β-oxidation of fatty acids and ω- and ω-1 hydroxylation of eicosanoids. Ethanol metabolism by Alcohol Dehydrogenase (ADH) increases the NADH/NAD^+^ ratio, inhibiting mitochondrial β-oxidation and contributing to alcohol-associated steatosis development. ADH has a lower Km for oxidation of hydroxylated fatty acid (10–40 μM) than for the oxidation of ethanol (Km 0.2–2 mM), indicating that high ethanol concentration (5.6 mM) will inhibit the oxidation of hydroxylated fatty acids [70]. In contrast, 17 μM ω-hydroxylated stearic acid inhibits oxidation of ethanol by ADH. Ethanol feeding increased fatty acid ω-oxidation in response to ethanol inhibition of mitochondrial β-oxidation [125]. *CYP2E1* has a higher Km for ethanol (8–10 mM) than ADH (0.2–2 mM) and thus metabolizes only 10% of body ethanol. Interestingly, rats fed a Lieber-DeCarli liquid ethanol diet supplemented with long-chain polyunsaturated fatty acid (PUFA) developed severe liver injury. In contrast, if the ethanol liquid diets were supplemented with medium-chain triglycerides, these rats displayed a normal liver [126]. Binge drinking induces adipose tissue lipolysis, increasing circulating free fatty acid (FFA) and enhancing *CYP2E1* activity, resulting in acute liver injury [127]. The synergistic effects of ethanol and increased serum fatty acid may be one mechanism of *CYP2E1*’s role in MASLD. In obese people with MASLD, increased *CYP2E1* protein content and activity correlated with the development of liver injury [128]. This contrasts the RNA-seq database analysis, in which *CYP2E1* mRNA decreased in MASH and levels dropped significantly in HCC. This difference may be due to increased serum FFAs in obese individuals compared to normal individuals with MASLD. Determining *CYP2E1* mRNA and protein levels at different stages of MASLD progression and *CYP2E1* activity will be necessary.

*CYP2E1* makes up 6.6% of total hepatic P450, while *CYP4A11* is less than 1% of total hepatic P450 under basal conditions [129], but their levels could be elevated by ethanol intake, ketone bodies, or fasting. It is apparent that the substrate, saturated fatty acid or arachidonic acid, and diet, high-fat or alcohol, determine the importance of *CYP2E1* ω-1 or *CYP4A11* ω-hydroxylation of these substrates in CLD. Liver disease induces macrovascular and microvascular events that result in microthrombi in the hepatic venules [130]. In cirrhotic patients, these microthrombi cause parenchymal extinction [131]. Microthrombi and parenchymal injury disrupt normal hepatic blood flow, causing hepatocyte apoptosis, which is replaced by fibrotic septa [132]. The recent observation that lung fibroblasts produce prostacyclin, which contributes to antithrombotic protection and blocks fibrosis, is a new paradigm [12]. It will be important to determine if *CYP2E1* 19-HETE activation of the prostacyclin receptor (IP) is protective in liver fibrosis and prevention of chronic liver disease progression.

## Conclusion

Chronic liver disease is characterized by portal hypertension, which is a driver of cirrhosis, ascites, gastro-esophageal varices, hepatorenal syndrome, hypersplenism, and hepatic encephalopathy due to portosystemic shunting. A large number of patients with portal hypertension have few therapeutic options. Currently, non-selective beta-blockers (NSBB) target the decrease of splanchnic venous inflow and cardiac output by blocking the β1-adrenergic receptor (β1-AR) and inducing vasoconstriction in the splanchnic region by blocking β2-AR. Unfortunately, NSBBs decrease portal pressure by only 15%, indicating that new therapies are still needed.

The *CYP4A11* ω-hydroxylase and the *CYP2E1* ω-1 hydroxylase have synergistic roles in the metabolism of saturated and unsaturated fatty acids during fasting or fatty acid overload in obesity and diabetes. Nuclear Erythroid Factor 2 (Nrf2) regulates the *CYP4A11* and *CYP2E1* genes during the stress response to fatty acids. The hydroxylation of fatty acids by ω and ω-1 hydroxylases and peroxisomal β-oxidation results in the production of succinate for gluconeogenesis and acetyl-CoA that can serve as a substrate for cholesterol and fatty acid synthesis, increasing lipogenesis and Lipid Droplet (LD) formation in the liver that results in steatosis. With the initiation of excess lipids in the liver, hepatitis ensues, and an inflammatory response results in the activation of phospholipase A2 and the release of Arachidonic Acid (AA). AA is ω-hydroxylated by *CYP4A11* and ω-1 hydroxylated by *CYP2E1*, producing 20-HETE and 19-HETE, respectively, that have opposing physiological roles in the progression of MASLD with *CYP4A11*-mediated 20-HETE vasoconstrictor activates the GPR75 receptor, which initiates inflammation and divergent un-characterized roles in the progression of MASLD. In contrast to the *CYP4A11*-mediated 20-HETE production, the *CYP2E1*-related ω-1 arachidonic acid metabolite 19-HETE is a vasodilator by activating the prostacyclin receptor, which opposes the vasoconstrictor function of 20-HETE. The decrease in *CYP2E1* during the progression of MASLD, as revealed by RNA-seq database analysis, suggests that the level of 19-HETE would decrease while the pro-lipogenic and inflammatory 20-HETE increases.

Many issues need to be addressed before determining the roles of the *CYP4A11*-mediated 20-HETE and *CYP2E1*-related 19-HETE in steatosis, steatohepatitis, cirrhosis, and HCC, and possible therapeutic targets in the treatment of CLD. Among these issues, 1) 19-HETE level needs to be determined during the progression of MASLD; 2) inhibition of COX1/2 seems to ameliorate the function of 20-HETE and thus the physiological role of 20-OH PGG_2_/PGH_2_ or 20-OH PGE_2_ needs to be determined; 3) what role does DCA play in the progression of MASLD, and 4) Can inhibition of the 20-HETE/GPR75 and activation of the 19-HETE/IP2 delay MASLD progression and be a therapeutic option in the treatment of portal hypertension in cirrhosis.

## Abbreviations

AA: Arachidonic Acid
ACAA1: Acetyl-CoA Acyltransferase 1
ACOX1: Acyl-CoA Oxidase
ACOT4: Acyl-CoA Thioesterase 4
ACOT8: Acyl-CoA Thioesterase 8
ACOT12: Acyl-CoA Thioesterase 12
ACE: Angiotensin-Converting Enzyme
ACLD: Acute on Chronic Liver Disease
ADH4: Alcohol Dehydrogenase 4
ALDH3A2: Aldehyde Dehydrogenase 3A2
ACSVL4: Acyl-CoA Synthetase Very Long Chain 4
ABCD3: ATP Binding Cassette Subfamily D Member 3
CD36: Cluster of Differentiation 36
CLD: Chronic Liver Disease
COX1/2: Cyclooxygenase 1/2
CYP4A11: Cytochrome P450 Family 4
CYP2E1: Cytochrome P450 2 Family
DCA: Dicarboxylic Acid
11,12 DiHETrE: 11,12-Dihydroxy-5Z,8Z,14Z-Eicosatrienoic Acid
ECM: Extracellular Matrix
EET: 2-Enoyl-CoA Hydratase, 3-Hydroxyacyl-CoA Dehydrogenase, EEHADH
EET: Epoxyeicosatrienoic Acid
EGFR: Epidermal Growth Factor Receptor
eNOS: Endothelial Nitric Oxide Synthase
FATP4: Fatty Acid Transport Protein 4
ERS: Endoplasmic Reticulum Stress
Km: Affinity Substrate for Enzyme
GPR75: G-Coupled Protein Receptor 75
FAO: Fatty Acid Oxidation
FFAR1: Free Fatty Acid Receptor 1
FFA: Free Fatty Acid
GSE: Gene-Expression Data Sets
HCC: Hepatocellular Carcinoma
HSC: Hepatic Stellate Cells
20-HETE: 20-Hydroxyeicosatetraenoic Acid
12-HHT: 12-(S)-Hydroxy-5Z,8E,10E-Heptadecatriaenoic Acid
HSD17B4: 17β-Hydroxysteroid Dehydrogenase 17B4
HODE: 13(S)-Hydroxy-9Z,11E-Octadecadienoic Acid
IL1: Interleukin 1
IL6: Interleukin 6
IPR: Prostacyclin Receptor
LRE: Linear Regression of Efficiency
LSEC: Liver Sinusoidal Endothelial Cells
MAPK: Mitogen-Activated Protein Kinase
MASH: Metabolic Dysfunction Steatosis Hepatitis
MASLD: Metabolic Dysfunction-Associated Steatotic Liver Disease
MCD: Methionine Choline-Deficient Diet
MRP2: Multi-Drug Resistance Associated Transport Protein 2
NAFLD: Non-Alcoholic Fatty Liver Disease
NASH: Non-Alcoholic Steatohepatitis
NEFA: Non-Esterified Fatty Acids
NF-κβ: Nuclear Factor Kappa-Light-Chain-Enhancer of Activated B Cells
NrF2: Nuclear Erythroid Factor 2
NSBB: Non-Selective Beta Blockers
PKA: Protein Kinase A
PGG2: Prostaglandin Endoperoxidase
PGH2: Prostaglandin Hydroquinone 2
PGE2: Prostaglandin E2
PVT: Portal Vein Thrombosis
RAS: Renin-Angiotensin System
SCPx: Sterol Carrier Protein X
TGF-β1: Transforming Growth Factor Beta 1
VEGF: Vascular Endothelial Growth Factor

## References

[1] Hardwick JP. Cytochrome P450 omega hydroxylase (CYP4) function in fatty acid metabolism and metabolic diseases. Biochem Pharmacol. 2008;75(12):2263–75. Available from: 10.1016/j.bcp.2008.03.004 18433732

[2] Garcia V, Shkolnik B, Milhau L, Falck JR, Schwartzman ML. 20-HETE activates the transcription of angiotensin-converting enzyme via nuclear factor-kappaB translocation and promoter binding. J Pharmacol Exp Ther. 2016;356(3):525–33. Available from: 10.1124/jpet.115.229377 26699146 PMC4767392

[3] Garcia V, Gilani A, Shkolnik B, Pandey V, Zhang FF, Dakarapu R, 20-HETE signals through G-protein-coupled receptor GPR75 (Gq) to affect vascular function and trigger hypertension. Circ Res. 2017;120(11):1776–88. Available from: 10.1161/circresaha.116.310525 28325781 PMC5446268

[4] Li B, Ma Y, Tan L, Ren H, Wu L, Su Q, 20-Hydroxytetraenoic acid induces hepatic fibrosis via the TGF-beta1/Smad3 signaling pathway. Toxicol Lett. 2023;373:1–12. Available from: 10.1016/j.toxlet.2022.11.001 36368619

[5] Akbari P, Gilani A, Sosina O, Kosmicki JA, Khrimian L, Fang YY, Sequencing of 640,000 exomes identifies GPR75 variants associated with protection from obesity. Science. 2021;373(6558). Available from: 10.1126/science.abf8683 PMC10275396 34210852

[6] Leeson-Payne C, Iyinikkel J, Malcolm C, Lam BYH, Sommer N, Dowsett GK, Loss of GPR75 protects against non-alcoholic fatty liver disease and body fat accumulation. Cell Metab. 2024;36(6):1076–87.e4. Available from: 10.1016/j.cmet.2024.03.016 38653246

[7] Yi X, Wu L, Liao D, Wang C, Zhang B. Interactions among CYP2C8, EPHX2, and CYP4A11 variants and CYP plasma metabolite levels in ischemic stroke. J Atheroscler Thromb. 2016;23(11):1286–93. Available from: 10.5551/jat.35279 27087514 PMC5065934

[8] Zhang X, Li S, Zhou Y, Su W, Ruan X, Wang B, Ablation of cytochrome P450 omega-hydroxylase 4A14 gene attenuates hepatic steatosis and fibrosis. Proc Natl Acad Sci U S A. 2017;114(12):3181–5. Available from: 10.1073/pnas.1700172114 28270609 PMC5373383

[9] Holla VR, Adas F, Imig JD, Zhao X, Price EJ Jr, Olsen N, Alterations in the regulation of androgen-sensitive Cyp4a monooxygenases cause hypertension. Proc Natl Acad Sci U S A. 2001;98(9):5211–6. Available from: 10.1073/pnas.081627898 11320253 PMC33189

[10] Laethem RM, Balazy M, Falck JR, Laethem CL, Koop DR. Formation of 19(S)-, 19(R)-, and 18(R)-hydroxyeicosatetraenoic acids by alcohol-inducible cytochrome P450 2E1. J Biol Chem. 1993;268(18):12912–8. Available from: https://pubmed.ncbi.nlm.nih.gov/8509425/8509425

[11] Tunaru S, Chennupati R, Nursing RM, Offermanns S. Arachidonic acid metabolite 19(S)-HETE induces vasorelaxation and platelet inhibition by activating prostacyclin (IP) receptor. PLoS One. 2016;11(10). Available from: 10.1371/journal.pone.0163633 PMC5035018 27662627

[12] Vinokurova M, Lopes-Pires ME, Cypaite N, Shala F, Armstrong PC, Ahmetaj-Shala B, Widening the prostacyclin paradigm: tissue fibroblasts are a critical site of production and antithrombotic protection. Arterioscler Thromb Vasc Biol. 2024;44(2):271–86. Available from: 10.1161/atvbaha.123.318923 37823267 PMC10749679

[13] Osmundsen H, Bremer J, Pedersen JI. Metabolic aspects of peroxisomal beta-oxidation. Biochim Biophys Acta. 1991;1085(2):141–58. Available from: 10.1016/0005-2760(91)90089-z 1892883

[14] Vamecq J, Draye JP. Peroxisomal and mitochondrial beta-oxidation of monocarboxylyl-CoA, omega-hydroxymonocarboxylyl-CoA and dicarboxylyl-CoA esters in tissues from untreated and clofibrate-treated rats. J Biochem. 1989;106(2):216–22. Available from: 10.1093/oxfordjournals.jbchem.a122835 2808318

[15] Vamecq J, Draye JP. Pathophysiology of peroxisomal beta-oxidation. Essays Biochem. 1989;24:115–225. Available from: https://pubmed.ncbi.nlm.nih.gov/2676523/2676523

[16] Wada F, Usami M. Studies on fatty acid omega-oxidation: antiketogenic effect and gluconeogenicity of dicarboxylic acids. Biochim Biophys Acta. 1977;487(3):361–8. Available from: https://pubmed.ncbi.nlm.nih.gov/861239/861239

[17] Adas F, Salaun JP, Berthou F, Picart D, Simon B, Amet Y. Requirement for omega and (omega-1)-hydroxylations of fatty acids by human cytochromes P450 2E1 and 4A11. J Lipid Res. 1999;40(10):1990–7. Available from: https://pubmed.ncbi.nlm.nih.gov/10553002/10553002

[18] Rizzo WB. Fatty aldehyde and fatty alcohol metabolism: review and importance for epidermal structure and function. Biochim Biophys Acta. 2014;1841(3):377–89. Available from: 10.1016/j.bbalip.2013.09.001 24036493 PMC3993971

[19] Hall AM, Wiczer BM, Herrmann T, Stremmel W, Bernlohr DA. Enzymatic properties of purified murine fatty acid transport protein 4 and analysis of acyl-CoA synthetase activities in tissues from FATP4 null mice. J Biol Chem. 2005;280(13):11948–54. Available from: 10.1074/jbc.m412629200 15653672

[20] Lu Y, George J. Interaction between fatty acid oxidation and ethanol metabolism in liver. Am J Physiol Gastrointest Liver Physiol. 2024;326(5). Available from: 10.1152/ajpgi.00281.2023 PMC11901390 38573193

[21] Ranea-Robles P, Chen H, Stauffer B, Yu C, Bhattacharya D, Friedman SL, The peroxisomal transporter ABCD3 plays a major role in hepatic dicarboxylic fatty acid metabolism and lipid homeostasis. J Inherit Metab Dis. 2021;44:1419–1433. Available from: 10.1002/jimd.12440 34564857 PMC8578467

[22] Ferdinandusse S, Denis S, Van Roermund CW, Wanders RJ, Dacremont G. Identification of the peroxisomal beta-oxidation enzymes involved in the degradation of long-chain dicarboxylic acids. J Lipid Res. 2004;45:1104–1111. Available from: 10.1194/jlr.m300512-jlr200 15060085

[23] Bergseth S, Hokland BM, Bremer J. Metabolism of dicarboxylic acids in vivo and in the perfused kidney of the rat. Biochim Biophys Acta. 1988;961:103–109. Available from: 10.1016/0005-2760(88)90135-x 3132982

[24] Suzuki H, Yamada J, Watanabe T, Suga T. Compartmentation of dicarboxylic acid beta-oxidation in rat liver: importance of peroxisomes in the metabolism of dicarboxylic acids. Biochim Biophys Acta. 1989;990:25–30. Available from: 10.1016/s0304-4165(89)80007-8 2914148

[25] Tserng KY, Griffin RL, Kerr DS. Distinction of dicarboxylic aciduria due to medium-chain triglyceride feeding from that due to abnormal fatty acid oxidation and fasting in children. Metabolism. 1996;45:162–167. Available from: 10.1016/s0026-0495(96)90047-5 8596483

[26] Jin Z, Bian F, Tomcik K, Kelleher JK, Zhang GF, Brunengraber H. Compartmentation of metabolism of the C12-, C9-, and C5-n-dicarboxylates in rat liver, investigated by mass isotopomer analysis: anaplerosis from dodecanedioate. J Biol Chem. 2015;290:18671–18677. Available from: 10.1074/jbc.m115.651737 26070565 PMC4513124

[27] Hunt MC, Rautanen A, Westin MA, Svensson LT, Alexson SE. Analysis of the mouse and human acyl-CoA thioesterase (ACOT) gene clusters shows that convergent, functional evolution results in a reduced number of human peroxisomal ACOTs. FASEB J. 2006;20:1855–1864. Available from: 10.1096/fj.06-6042com 16940157

[28] Westin MA, Hunt MC, Alexson SE. The identification of a succinyl-CoA thioesterase suggests a novel pathway for succinate production in peroxisomes. J Biol Chem. 2005;280:38125–38132. Available from: 10.1074/jbc.m508479200 16141203

[29] Mortensen PB. The possible antiketogenic and gluconeogenic effect of the omega-oxidation of fatty acids in rats. Biochim Biophys Acta. 1980;620:177–185. Available from: 10.1016/0005-2760(80)90199-x 7437451

[30] Leighton F, Bergseth S, Rortveit T, Christiansen EN, Bremer J. Free acetate production by rat hepatocytes during peroxisomal fatty acid and dicarboxylic acid oxidation. J Biol Chem. 1989;264:10347–10350. Available from: https://pubmed.ncbi.nlm.nih.gov/2732225/2732225

[31] Wang Y, Zhang L, Gai Y, He H, Qiu P, Li P. Identification of key biomarkers in hepatocellular carcinoma induced by non-alcoholic steatohepatitis or metabolic syndrome via integrated bioinformatics analysis. Cell Mol Biol (Noisy-le-Grand). 2023;69:174–180. Available from: 10.14715/cmb/2023.69.7.28 37715392

[32] Leahy C, Osborne N, Shirota L, Rote P, Lee YK, Song BJ, The fatty acid omega hydroxylase genes (CYP4 family) in the progression of metabolic dysfunction-associated steatotic liver disease (MASLD): an RNA sequence database analysis and review. Biochem Pharmacol. 2024;116241. Available from: 10.1016/j.bcp.2024.116241 38697309 PMC11774579

[33] Zhang X, Gao T, Deng S, Shang L, Chen X, Chen K, Fasting induces hepatic lipid accumulation by stimulating peroxisomal dicarboxylic acid oxidation. J Biol Chem. 2021;296:100622. Available from: 10.1016/j.jbc.2021.100622 33811861 PMC8102918

[34] Dietschy JM, Brown MS. Effect of alterations of the specific activity of the intracellular acetyl CoA pool on apparent rates of hepatic cholesterogenesis. J Lipid Res. 1974;15:508–516. Available from: https://pubmed.ncbi.nlm.nih.gov/4413018/4413018

[35] Passi S, Picardo M, Nazzaro-Porro M, Breathnach A, Confaloni AM, Serlupi-Crescenzi G. Antimitochondrial effect of saturated medium chain length (C8-C13) dicarboxylic acids. Biochem Pharmacol. 1984;33:103–108. Available from: 10.1016/0006-2952(84)90376-9 6704136

[36] Tonsgard JH. Urinary dicarboxylic acids in Reye syndrome. J Pediatr. 1985;107:79–84. Available from: 10.1016/s0022-3476(85)80619-3 4009343

[37] RungratanawanichW, BallwayJW, WangX, WonKJ, HardwickJP, SongBJ. Post-translational modifications of histone and non-histone proteins in epigenetic regulation and translational applications in alcohol-associated liver disease: challenges and research opportunities. Pharmacol Ther. 2023;251:108547. Available from: 10.1016/j.pharmthera.2023.10854737838219

[38] Inouye M, Mio T, Sumino K. Dicarboxylic acids as markers of fatty acid peroxidation in diabetes. Atherosclerosis. 2000;148:197–202. Available from: 10.1016/s0021-9150(99)00263-4 10580186

[39] Lu D, He A, Tan M, Mrad M, El Daibani A, Hu D, Liver ACOX1 regulates levels of circulating lipids that promote metabolic health through adipose remodeling. Nat Commun. 2024;15:4214. Available from: https://www.nature.com/articles/s41467-024-48471-2 38760332 10.1038/s41467-024-48471-2 PMC11101658

[40] Abdelmegeed MA, Banerjee A, Jang S, Yoo SH, Yun JW, Gonzalez FJ, CYP2E1 potentiates binge alcohol-induced gut leakiness, steatohepatitis, and apoptosis. Free Radic Biol Med. 2013;65:1238–1245. Available from: 10.1016/j.freeradbiomed.2013.09.009 24064383 PMC3859835

[41] Ruiz-Sala P, Pena-Quintana L. Biochemical markers for the diagnosis of mitochondrial fatty acid oxidation diseases. J Clin Med. 2021;10:4855. Available from: 10.3390/jcm10214855 34768374 PMC8584803

[42] Baldi S, Menicatti M, Nannini G, Niccolai E, Russo E, Ricci F, Free fatty acids signature in human intestinal disorders: significant association between butyric acid and celiac disease. Nutrients. 2021;13:742. Available from: 10.3390/nu13030742 33652681 PMC7996737

[43] Mingrone G, Castagneto-Gissey L, Mace K. Use of dicarboxylic acids in type 2 diabetes. Br J Clin Pharmacol. 2013;75:671–676. Available from: 10.1111/j.1365-2125.2012.04177.x 22242741 PMC3575934

[44] Butera E, Termite F, Esposto G, Galasso L, Mignini I, Borriello R, Exploring the role of bempedoic acid in metabolic dysfunction associated steatotic liver disease: actual evidence and future perspectives. Int J Mol Sci. 2024;25:6938. Available from: 10.3390/ijms25136938 39000046 PMC11241610

[45] Goetzman ES, Zhang BB, Zhang Y, Bharathi SS, Bons J, Rose J, Dietary dicarboxylic acids provide a non-storable alternative fat source that protects mice against obesity. J Clin Invest. 2024;134:e174186. Available from: 10.1172/jci174186 38687608 PMC11178532

[46] Dias VC, Fung E, Snyder FF, Carter RJ, Parsons HG. Effects of medium-chain triglyceride feeding on energy balance in adult humans. Metabolism. 1990;39:887–891. Available from: 10.1016/0026-0495(90)90295-n 2392059

[47] Wang H, Lu J, Chen X, Schwalbe M, Gorka JE, Mandel JA, Acquired deficiency of peroxisomal dicarboxylic acid catabolism is a metabolic vulnerability in hepatoblastoma. J Biol Chem. 2021;296:100283. Available from: 10.1016/j.jbc.2021.100283 33450224 PMC7948956

[48] Gao H, Cao Y, Xia H, Zhu X, Jin Y. CYP4A11 is involved in the development of nonalcoholic fatty liver disease via ROS-induced lipid peroxidation and inflammation. Int J Mol Med. 2020;45:1121–1129. Available from: 10.3892/ijmm.2020.4479 32124935 PMC7053872

[49] Ni KD, Liu JY. The functions of cytochrome P450 omega-hydroxylases and the associated eicosanoids in inflammation-related diseases. Front Pharmacol. 2021;12:716801. Available from: 10.3389/fphar.2021.716801 34594219 PMC8476763

[50] Trefely S, Lovell CD, Snyder NW, Wellen KE. Compartmentalised acyl-CoA metabolism and roles in chromatin regulation. Mol Metab. 2020;38:100941. Available from: 10.1016/j.molmet.2020.01.005 32199817 PMC7300382

[51] Lu Y, Travnickova J, Badonyi M, Rambow F, Coates A, Khan Z, ALDH1A3-acetaldehyde metabolism potentiates transcriptional heterogeneity in melanoma. Cell Rep. 2024;43:114406. Available from: 10.1016/j.celrep.2024.114406 38963759 PMC11290356

[52] Hardwick JPS, Rote P, Leahy C, Lee YK, Wolf AR, Diegisser D, The CYP4/20-HETE/GPR75 axis in the progression of metabolic-associated steatosis liver disease (MASLD) to chronic liver disease. Front Physiol. 2024. 10.3389/fphys.2024.1497297 PMC11826315 39959811

[53] Gilani A, Agostinucci K, Hossain S, Pascale JV, Garcia V, Adebesin AM, 20-HETE interferes with insulin signaling and contributes to obesity-driven insulin resistance. Prostaglandins Other Lipid Mediat. 2021;152:106485. Available from: 10.1016/j.prostaglandins.2020.106485 33011364 PMC7855891

[54] Li S, Wang C, Zhang X, Su W. Cytochrome P450 Omega-Hydroxylase 4a14 attenuates cholestatic liver fibrosis. Front Physiol. 2021;12:688259. Available from: 10.3389/fphys.2021.688259 34135776 PMC8201794

[55] Muller DN, Schmidt C, Barbosa-Sicard E, Wellner M, Gross V, Hercule H, Mouse Cyp4a isoforms: enzymatic properties, gender- and strain-specific expression, and role in renal 20-hydroxyeicosatetraenoic acid formation. Biochem J. 2007;403:109–118. Available from: 10.1042/bj20061328 17112342 PMC1828894

[56] Nelson DR, Zeldin DC, Hoffman SM, Maltais LJ, Wain HM, Nebert DW. Comparison of cytochrome P450 (CYP) genes from the mouse and human genomes, including nomenclature recommendations for genes, pseudogenes, and alternative-splice variants. Pharmacogenetics. 2004;14:1–18. Available from: 10.1097/00008571-200401000-00001 15128046

[57] Song BJ. Ethanol-inducible cytochrome P450 (CYP2E1): biochemistry, molecular biology and clinical relevance: 1996 update. Alcohol Clin Exp Res. 1996;20:138A–146A. Available from: 10.1111/j.1530-0277.1996.tb01764.x 8947253

[58] Song BJ, Abdelmegeed MA, Cho YE, Akbar M, Rhim JS, Song MK, Contributing roles of CYP2E1 and other cytochrome P450 isoforms in alcohol-related tissue injury and carcinogenesis. Adv Exp Med Biol. 2019;1164:73–87. Available from: 10.1007/978-3-030-22254-3_6 31576541

[59] Daly AK. Relevance of CYP2E1 to non-alcoholic fatty liver disease. Subcell Biochem. 2013;67:165–175. Available from: 10.1007/978-94-007-5881-0_5 23400921

[60] Abdelmegeed MA, Banerjee A, Yoo SH, Jang S, Gonzalez FJ, Song BJ. Critical role of cytochrome P450 2E1 (CYP2E1) in the development of high fat-induced non-alcoholic steatohepatitis. J Hepatol. 2012;57:860–866. Available from: 10.1016/j.jhep.2012.05.019 22668639 PMC3445664

[61] Das SK, Vasudevan DM. Alcohol-induced oxidative stress. Life Sci. 2007;81:177–187. Available from: 10.1016/j.lfs.2007.05.005 17570440

[62] Porubsky PR, Meneely KM, Scott EE. Structures of human cytochrome P-450 2E1: insights into the binding of inhibitors and both small molecular weight and fatty acid substrates. J Biol Chem. 2008;283:33698–707. Available from: 10.1074/jbc.m805999200 18818195 PMC2586265

[63] Dostalek M, Brooks JD, Hardy KD, Milne GL, Moore MM, Sharma S, In vivo oxidative damage in rats is associated with barbiturate response but not other cytochrome P450 inducers. Mol Pharmacol. 2007;72:1419–1424. Available from: 10.1124/mol.107.040238 17898314

[64] Bansal S, Liu CP, Sepuri NB, Anandatheerthavarada HK, Selvaraj V, Hoek J, Mitochondria-targeted cytochrome P450 2E1 induces oxidative damage and augments alcohol-mediated oxidative stress. J Biol Chem. 2010;285:24609–24619. Available from: 10.1074/jbc.m110.121822 20529841 PMC2915697

[65] Massart J, Begriche K, Hartman JH, Fromenty B. Role of mitochondrial cytochrome P450 2E1 in healthy and diseased liver. Cells. 2022;11:288. Available from: 10.3390/cells11020288 35053404 PMC8774478

[66] Bai J, Cederbaum AI. Overexpression of CYP2E1 in mitochondria sensitizes HepG2 cells to the toxicity caused by depletion of glutathione. J Biol Chem. 2006;281:5128–5136. Available from: 10.1074/jbc.m510484200 16380384

[67] Bansal S, Anandatheerthavarada HK, Prabu GK, Milne GL, Martin MV, Guengerich FP, Human cytochrome P450 2E1 mutations that alter mitochondrial targeting efficiency and susceptibility to ethanol-induced toxicity in cellular models. J Biol Chem. 2013;288:12627–12644. Available from: 10.1074/jbc.m113.452367 23471973 PMC3642310

[68] Amet Y, Adas F, Nanji AA. Fatty acid omega- and (omega-1)-hydroxylation in experimental alcoholic liver disease: relationship to different dietary fatty acids. Alcohol Clin Exp Res. 1998;22:1493–1500. Available from: https://pubmed.ncbi.nlm.nih.gov/9802534/9802534

[69] Amet Y, Berthou F, Baird S, Dreano Y, Bail JP, Menez JF. Validation of the (omega-1)-hydroxylation of lauric acid as an in vitro substrate probe for human liver CYP2E1. Biochem Pharmacol. 1995;50:1775–1782. Available from: 10.1016/0006-2952(95)02040-3 8615855

[70] Bjorkhem I On the role of alcohol dehydrogenase in omega-oxidation of fatty acids. Eur J Biochem. 1972;30:441–451. Available from: 10.1111/j.1432-1033.1972.tb02116.x 4565405

[71] Keller MA, Watschinger K, Golderer G, Maglione M, Sarg B, Lindner HH, Monitoring of fatty aldehyde dehydrogenase by formation of pyrenedecanoic acid from pyrenedecanal. J Lipid Res. 2010;51:1554–1559. Available from: 10.1194/jlr.d002220 19965611 PMC3035519

[72] Zhou J, Yin Y, Yang Y, Peng D, Wei J, Yin G, Knockdown of miR-423–5p simultaneously upgrades the eNOS and VEGFa pathways in ADSCs and improves erectile function in diabetic rats. J Cell Mol Med. 2021;25:9796–9804. Available from: 10.1111/jcmm.16927 34545676 PMC8505849

[73] Deleve LD. Sinusoidal obstruction syndrome. Gastroenterol Hepatol (N Y). 2008;4:101–103. Available from: https://pubmed.ncbi.nlm.nih.gov/21904484/21904484 PMC3088834

[74] Funyu J, Mochida S, Inao M, Matsui A, Fujiwara K. VEGF can act as vascular permeability factor in the hepatic sinusoids through upregulation of porosity of endothelial cells. Biochem Biophys Res Commun. 2001;280:481–485. Available from: 10.1006/bbrc.2000.4148 11162543

[75] Iwakiri Y, Trebicka J. Portal hypertension in cirrhosis: pathophysiological mechanisms and therapy. JHEP Rep. 2021;3:100316. Available from: 10.1016/j.jhepr.2021.100316 34337369 PMC8318926

[76] Scroggins BT, Robzyk K, Wang D, Marcu MG, Tsutsumi S, Beebe K, An acetylation site in the middle domain of Hsp90 regulates chaperone function. Mol Cell. 2007;25:151–159. Available from: 10.1016/j.molcel.2006.12.008 17218278 PMC1839984

[77] Schierwagen R, Dietrich P, Klein S, Uschner FE, Ortiz C, Tyc O, beta-Arrestin2 is increased in liver fibrosis in humans and rodents. Proc Natl Acad Sci U S A. 2020;117:27082–27084. Available from: 10.1073/pnas.2014337117 33144522 PMC7959505

[78] Attal N, Marrero E, Thompson KJ, McKillop IH. Cytochrome P450 2E1-dependent hepatic ethanol metabolism induces fatty acid-binding protein 4 and steatosis. Alcohol Clin Exp Res. 2022;46:928–940. Available from: 10.1111/acer.14828 35403271 PMC9246908

[79] Gao N, Chen J, Li Y, Ding Y, Han Z, Xu H, The CYP2E1 inhibitor Q11 ameliorates LPS-induced sepsis in mice by suppressing oxidative stress and NLRP3 activation. Biochem Pharmacol. 2023;214:115638. Available from: 10.1016/j.bcp.2023.115638 37290597

[80] Nagappan A, Jung DY, Kim JH, Lee H, Jung MH. Gomisin N alleviates ethanol-induced liver injury through ameliorating lipid metabolism and oxidative stress. Int J Mol Sci. 2018;19:2601. Available from: 10.3390/ijms19092601 30200508 PMC6164513

[81] Zong H, Armoni M, Harel C, Karnieli E, Pessin JE. Cytochrome P-450 CYP2E1 knockout mice are protected against high-fat diet-induced obesity and insulin resistance. Am J Physiol Endocrinol Metab. 2012;302:E532–539. Available from: 10.1152/ajpendo.00258.2011 22185839 PMC3311288

[82] Woodcroft KJ, Hafner MS, Novak RF. Insulin signaling in the transcriptional and posttranscriptional regulation of CYP2E1 expression. Hepatology. 2002;35:263–273. Available from: 10.1053/jhep.2002.30691 11826398

[83] Kathirvel E, Morgan K, French SW, Morgan TR. Overexpression of liver-specific cytochrome P4502E1 impairs hepatic insulin signaling in a transgenic mouse model of nonalcoholic fatty liver disease. Eur J Gastroenterol Hepatol. 2009;21:973–983. Available from: 10.1097/meg.0b013e328328f461 19307976

[84] Leclercq IA, Farrell GC, Field J, Bell DR, Gonzalez FJ, Robertson GR. CYP2E1 and CYP4A as microsomal catalysts of lipid peroxides in murine nonalcoholic steatohepatitis. J Clin Invest. 2000;105:1067–1075. Available from: 10.1172/jci8814 10772651 PMC300833

[85] Park EC, Kim SI, Hong Y, Hwang JW, Cho GS, Cha HN, Inhibition of CYP4A reduces hepatic endoplasmic reticulum stress and features of diabetes in mice. Gastroenterology. 2014;147:860–869. Available from: 10.1053/j.gastro.2014.06.039 24983671

[86] Gilani A, Pandey V, Garcia V, Agostinucci K, Singh SP, Schragenheim J, High-fat diet-induced obesity and insulin resistance in CYP4a14(−/−) mice is mediated by 20-HETE. Am J Physiol Regul Integr Comp Physiol. 2018;315:R934–944. Available from: 10.1152/ajpregu.00125.2018 30088983 PMC6295494

[87] Gong P, Cederbaum AI. Transcription factor Nrf2 protects HepG2 cells against CYP2E1 plus arachidonic acid-dependent toxicity. J Biol Chem. 2006;281:14573–14579. Available from: 10.1074/jbc.m600613200 16551616

[88] Tice AL, Steiner JL. Binge alcohol induces NRF2-related antioxidant response in the skeletal muscle of female mice. Biochem Biophys Res Commun. 2024;714:149968. Available from: 10.1016/j.bbrc.2024.149968 38657445

[89] Ma Q Role of Nrf2 in oxidative stress and toxicity. Annu Rev Pharmacol Toxicol. 2013;53:401–426. Available from: 10.1146/annurev-pharmtox-011112-140320 23294312 PMC4680839

[90] Meakin PJ, Chowdhry S, Sharma RS, Ashford FB, Walsh SV, McCrimmon RJ, Susceptibility of Nrf2-null mice to steatohepatitis and cirrhosis upon consumption of a high-fat diet is associated with oxidative stress, perturbation of the unfolded protein response, and disturbance in the expression of metabolic enzymes but not with insulin resistance. Mol Cell Biol. 2014;34:3305–3320. Available from: 10.1128/mcb.00677-14 24958099 PMC4135558

[91] Tunaru S, Bonnavion R, Brandenburger I, Preussner J, Thomas D, Scholich K, 20-HETE promotes glucose-stimulated insulin secretion in an autocrine manner through FFAR1. Nat Commun. 2018;9:177. Available from: 10.1038/s41467-017-02539-4 29330456 PMC5766607

[92] Wu CC, Gupta T, Garcia V, Ding Y, Schwartzman ML. 20-HETE and blood pressure regulation: clinical implications. Cardiol Rev. 2014;22:1–12. Available from: 10.1097/crd.0b013e3182961659 23584425 PMC4292790

[93] Froogh G, Garcia V, Laniado Schwartzman M. The CYP/20-HETE/GPR75 axis in hypertension. Adv Pharmacol. 2022;94:1–25. Available from: 10.1016/bs.apha.2022.02.003 35659370 PMC10123763

[94] Helal SA, Gerges SH, El-Kadi AOS. Enantioselectivity in some physiological and pathophysiological roles of hydroxyeicosatetraenoic acids. Drug Metab Rev. 2024;56:31–45. Available from: 10.1080/03602532.2023.2284110 38358327

[95] Panigrahy D, Kaipainen A, Greene ER, Huang S. Cytochrome P450-derived eicosanoids: the neglected pathway in cancer. Cancer Metastasis Rev. 2010;29:723–735. Available from: 10.1007/s10555-010-9264-x 20941528 PMC2962793

[96] Schuck RN, Theken KN, Edin ML, Caughey M, Bass A, Ellis K, Cytochrome P450-derived eicosanoids and vascular dysfunction in coronary artery disease patients. Atherosclerosis. 2013;227:442–448. Available from: 10.1016/j.atherosclerosis.2013.01.034 23466098 PMC3638946

[97] Dakarapu R, Errabelli R, Manthati VL, Adebesin AM, Barma DK, Barma D, 19-Hydroxyeicosatetraenoic acid analogs: Antagonism of 20-hydroxyeicosatetraenoic acid-induced vascular sensitization and hypertension. Bioorg Med Chem Lett. 2019;29:126616. Available from: 10.1016/j.bmcl.2019.08.020 31439380 PMC6745249

[98] Shoieb SM, El-Sherbeni AA, El-Kadi AOS. Subterminal hydroxy eicosatetraenoic acids: Crucial lipid mediators in normal physiology and disease states. Chem Biol Interact. 2019;299:140–150. Available from: 10.1016/j.cbi.2018.12.004 30543782

[99] Powell PK, Wolf I, Lasker JM. Identification of CYP4A11 as the major lauric acid omega-hydroxylase in human liver microsomes. Arch Biochem Biophys. 1996;335:219–226. Available from: 10.1006/abbi.1996.0501 8914854

[100] Porubsky PR, Battaile KP, Scott EE. Human cytochrome P450 2E1 structures with fatty acid analogs reveal a previously unobserved binding mode. J Biol Chem. 2010;285:22282–22290. Available from: 10.1074/jbc.m110.109017 20463018 PMC2903405

[101] Pascale JV, Park EJ, Adebesin AM, Falck JR, Schwartzman ML, Garcia V. Uncovering the signalling, structure, and function of the 20-HETE-GPR75 pairing: Identifying the chemokine CCL5 as a negative regulator of GPR75. Br J Pharmacol. 2021;178:3813–3828. Available from: 10.1111/bph.15525 33974269 PMC10119890

[102] Escalante B, Falck JR, Yadagiri P, Sun LM, Laniado-Schwartzman M. 19(S)-hydroxyeicosatetraenoic acid is a potent stimulator of renal Na+-K+-ATPase. Biochem Biophys Res Commun. 1988;152:1269–1274. Available from: 10.1016/s0006-291x(88)80422-4 2837181

[103] Escalante B, Sessa WC, Falck JR, Yadagiri P, Schwartzman ML. Cytochrome P450-dependent arachidonic acid metabolites, 19- and 20-hydroxyeicosatetraenoic acids, enhance sodium-potassium ATPase activity in vascular smooth muscle. J Cardiovasc Pharmacol. 1990;16:438–443. Available from: 10.1097/00005344-199009000-00013 1700215

[104] Hammoutene A, Rautou PE. Role of liver sinusoidal endothelial cells in non-alcoholic fatty liver disease. J Hepatol. 2019;70:1278–1291. Available from: 10.1016/j.jhep.2019.02.012 30797053

[105] Ruan B, Duan JL, Xu H, Tao KS, Han H, Dou GR, Capillarized liver sinusoidal endothelial cells undergo partial endothelial-mesenchymal transition to actively deposit sinusoidal ECM in liver fibrosis. Front Cell Dev Biol. 2021;9:671081. Available from: 10.3389/fcell.2021.671081 34277612 PMC8285099

[106] Leung TM, Tipoe GL, Liong EC, Lau TY, Fung ML, Nanji AA. Endothelial nitric oxide synthase is a critical factor in experimental liver fibrosis. Int J Exp Pathol. 2008;89:241–250. Available from: 10.1111/j.1365-2613.2008.00590.x 18429990 PMC2525781

[107] Hartl L, Rumpf B, Domenig O, Simbrunner B, Paternostro R, Jachs M, The systemic and hepatic alternative renin-angiotensin system is activated in liver cirrhosis, linked to endothelial dysfunction and inflammation. Sci Rep. 2023;13:953. Available from: 10.1038/s41598-023-28239-2 36653504 PMC9849268

[108] Bataller R, Nicolas JM, Gines P, Gorbig MN, Garcia-Ramallo E, Lario S, Contraction of human hepatic stellate cells activated in culture: a role for voltage-operated calcium channels. J Hepatol. 1998;29:398–408. Available from: 10.1016/s0168-8278(98)80057-3 9764986

[109] Bataller R, Gines P, Nicolas JM, Gorbig MN, Garcia-Ramallo E, Gasull X, Angiotensin II induces contraction and proliferation of human hepatic stellate cells. Gastroenterology. 2000;118:1149–1156. Available from: 10.1016/s0016-5085(00)70368-4 10833490

[110] Sacerdoti D, Balazy M, Angeli P, Gatta A, McGiff JC. Eicosanoid excretion in hepatic cirrhosis. Predominance of 20-HETE. J Clin Invest. 1997;100:1264–1270. Available from: 10.1172/jci119640 9276745 PMC508304

[111] Sacerdoti D, Jiang H, Gaiani S, McGiff JC, Gatta A, Bolognesi M. 11,12-EET increases porto-sinusoidal resistance and may play a role in endothelial dysfunction of portal hypertension. Prostaglandins Other Lipid Mediat. 2011;96:72–75. Available from: 10.1016/j.prostaglandins.2011.08.002 21856435 PMC4540347

[112] Sacerdoti D, Pesce P, Di Pascoli M, Brocco S, Cecchetto L, Bolognesi M. Arachidonic acid metabolites and endothelial dysfunction of portal hypertension. Prostaglandins Other Lipid Mediat. 2015;120:80–90. Available from: 10.1016/j.prostaglandins.2015.05.008 26072731

[113] Schwartzman ML, Falck JR, Yadagiri P, Escalante B. Metabolism of 20-hydroxyeicosatetraenoic acid by cyclooxygenase. Formation and identification of novel endothelium-dependent vasoconstrictor metabolites. J Biol Chem. 1989;264:11658–11662. Available from: https://pubmed.ncbi.nlm.nih.gov/2501294/2501294

[114] Zhang Y, Hoda MN, Zheng X, Li W, Luo P, Maddipati KR, Combined therapy with COX-2 inhibitor and 20-HETE inhibitor reduces colon tumor growth and the adverse effects of ischemic stroke associated with COX-2 inhibition. Am J Physiol Regul Integr Comp Physiol. 2014;307:R693–703. Available from: 10.1152/ajpregu.00422.2013 24990856 PMC4214836

[115] Liu JY, Li N, Yang J, Li N, Qiu H, Ai D, Metabolic profiling of murine plasma reveals an unexpected biomarker in rofecoxib-mediated cardiovascular events. Proc Natl Acad Sci U S A. 2010;107:17017–17022. Available from: 10.1073/pnas.1011278107 20837537 PMC2947894

[116] Sacerdoti D, Gatta A, McGiff JC. Role of cytochrome P450-dependent arachidonic acid metabolites in liver physiology and pathophysiology. Prostaglandins Other Lipid Mediat. 2003;72:51–71. Available from: 10.1016/s1098-8823(03)00077-7 14626496

[117] Kim DH, Puri N, Sodhi K, Falck JR, Abraham NG, Shapiro J, Cyclooxygenase-2 dependent metabolism of 20-HETE increases adiposity and adipocyte enlargement in mesenchymal stem cell-derived adipocytes. J Lipid Res. 2013;54:786–793. Available from: 10.1194/jlr.m033894 23293373 PMC3617952

[118] Alexanian A, Miller B, Roman RJ, Sorokin A. 20-HETE-producing enzymes are up-regulated in human cancers. Cancer Genomics Proteomics. 2012;9:163–169. Available from: https://pubmed.ncbi.nlm.nih.gov/22798501/22798501 PMC3601443

[119] Gu W, Zeng D, Zhang C. Discovering the effect of combination of celecoxib and sorafenib on hepatocellular carcinoma. Discov Oncol. 2024;15:321. Available from: 10.1007/s12672-024-01203-w 39083127 PMC11291820

[120] Morisaki T, Umebayashi M, Kiyota A, Koya N, Tanaka H, Onishi H, Combining celecoxib with sorafenib synergistically inhibits hepatocellular carcinoma cells in vitro. Anticancer Res. 2013;33:1387–1395. Available from: https://pubmed.ncbi.nlm.nih.gov/23564777/23564777

[121] Cui J, Guo YH, Zhang HY, Jiang LL, Ma JQ, Wang WJ, Cyclooxygenase-2 inhibitor is a robust enhancer of anticancer agents against hepatocellular carcinoma multicellular spheroids. Onco Targets Ther. 2014;7:353–363. Available from: 10.2147/ott.s56115 24591842 PMC3938498

[122] Hsu MH, Savas U, Griffin KJ, Johnson EF. Regulation of human cytochrome P450 4F2 expression by sterol regulatory element-binding protein and lovastatin. J Biol Chem. 2007;282:5225–5236. Available from: Available from: 10.1074/jbc.m608176200 17142457

[123] Poloyac SM, Tortorici MA, Przychodzin DI, Reynolds RB, Xie W, Frye RF, The effect of isoniazid on CYP2E1- and CYP4A-mediated hydroxylation of arachidonic acid in the rat liver and kidney. Drug Metab Dispos. 2004;32:727–733. Available from: 10.1124/dmd.32.7.727 15205388

[124] Zhang Q, Wu S, Chen Q, Zhang Y, Zhang C, Yin R, Reducing oxidative stress-mediated alcoholic liver injury by multiplexed RNAi of Cyp2e1, Cyp4a10, and Cyp4a14. Biomedicines. 2024;12:1505. Available from: 10.3390/biomedicines12071505 39062078 PMC11274525

[125] Ma X, Baraona E, Lieber CS. Alcohol consumption enhances fatty acid omega-oxidation, with a greater increase in male than in female rats. Hepatology. 1993;18:1247–1253. Available from: https://pubmed.ncbi.nlm.nih.gov/8225232/8225232

[126] Polavarapu R, Spitz DR, Sim JE, Follansbee MH, Oberley LW, Rahemtulla A, Increased lipid peroxidation and impaired antioxidant enzyme function is associated with pathological liver injury in experimental alcoholic liver disease in rats fed diets high in corn oil and fish oil. Hepatology. 1998;27:1317–1323. Available from: 10.1002/hep.510270518 9581686

[127] Malnick SDH, Alin P, Somin M, Neuman MG. Fatty liver disease—alcoholic and non-alcoholic: similar but different. Int J Mol Sci. 2022;23:1626. Available from: 10.3390/ijms232416226 36555867 PMC9783455

[128] Orellana M, Rodrigo R, Varela N, Araya J, Poniachik J, Csendes A, Relationship between in vivo chlorzoxazone hydroxylation, hepatic cytochrome P450 2E1 content and liver injury in obese non-alcoholic fatty liver disease patients. Hepatol Res. 2006;34:57–63. Available from: 10.1016/j.hepres.2005.10.001 16321567

[129] Michaels S, Wang MZ. The revised human liver cytochrome P450 “Pie”: absolute protein quantification of CYP4F and CYP3A enzymes using targeted quantitative proteomics. Drug Metab Dispos. 2014;42:1241–1251. Available from: 10.1124/dmd.114.058040 24816681 PMC4109210

[130] Northup PG, Argo CK, Shah N, Caldwell SH. Hypercoagulation and thrombophilia in nonalcoholic fatty liver disease: mechanisms, human evidence, therapeutic implications, and preventive implications. Semin Liver Dis. 2012;32:39–48. Available from: 10.1055/s-0032-1306425 22418887

[131] Anstee QM, Wright M, Goldin R, Thursz MR. Parenchymal extinction: coagulation and hepatic fibrogenesis. Clin Liver Dis. 2009;13:117–126. Available from: 10.1016/j.cld.2008.09.013 19150316

[132] Wanless IR, Wong F, Blendis LM, Greig P, Heathcote EJ, Levy G. Hepatic and portal vein thrombosis in cirrhosis: possible role in the development of parenchymal extinction and portal hypertension. Hepatology. 1995;21:1238–1247. Available from: https://pubmed.ncbi.nlm.nih.gov/7737629/7737629

